# Posterior approaches to the acetabulum

**DOI:** 10.1007/s00402-024-05583-0

**Published:** 2024-09-26

**Authors:** Mario Staresinic, Richard A. Lindtner, Dietmar Krappinger, Axel Gänsslen

**Affiliations:** 1 Clinic for Surgery, Department of General and Sports Traumatology, University Hospital “Merkur” Zagreb, Zagreb, Croatia; 2 Department of Orthopaedics and Traumatology, Medical University of Innsbruck, Anichstraße 35, Innsbruck, 6020 Austria; 3 Trauma Department, Hannover Medical School, Hannover, Germany; 4 Department of Trauma and Orthopedics, Johannes Wesling Hospital, Minden, Germany

**Keywords:** Acetabulum, Hip, Acetabular fracture, Surgical approach, Posterior approach, Kocher-Langenbeck approach, Gibson approach, Modified Gibson approach, Ganz approach, Trochanteric osteotomy, Surgical hip dislocation

## Abstract

Posterior approaches, particularly the Kocher-Langenbeck approach, remain the workhorses in the treatment of acetabular fractures. Various modifications have been developed, each offering specific advantages depending on surgical requirements. The modified Gibson approach, for example, is suggested to provide enhanced visualization of the superior acetabulum, although recent cadaveric studies have not consistently substantiated this benefit. The Ganz approach, which involves bigastric trochanteric osteotomy with safe surgical hip dislocation, is particularly advantageous for managing complex and comminuted posterior acetabular fractures, as it enables a 360° view of the acetabulum and femoral head. Overall, posterior approaches are associated with low rates of complications, with heterotopic ossification being the most prevalent. The choice of surgical approach and patient positioning should be guided by the surgeon’s preference and expertise, tailored to the specific fracture pattern and patient characteristics.

## Introduction

Following the standardization of treatment for displaced acetabular fractures through open reduction and internal fixation (ORIF), historically, only a limited number of standard surgical approaches were employed, including the Kocher-Langenbeck, ilioinguinal, and extended iliofemoral approaches [20]. With increasing surgical experience in the 1980s and 1990s, several modifications to these approaches were developed to address specific fracture scenarios. Notably, the extended iliofemoral approach as described by Reinert [34] (commonly referred to as Baltimore approach) and the triradiate approach as described by Mears [25] were introduced. However, these approaches are rarely utilized due to the extensive soft tissue dissection required and the higher complication rates [51], compared to single approaches.

Alternatively, the combination of an anterior and posterior standard approach has been recommended [11, 36, 40]. However, this strategy presents drawbacks such as prolonged operative time and increased blood loss without demonstrating superior outcomes compared to single approach techniques.

For an extended period, the posterior Kocher-Langenbeck approach and the anterior ilioinguinal approach were the predominant approaches used for the majority of patients [20]. These single-column approaches, which allow for visualization of one acetabular column, remain the preferred options in clinical practice [15, 43].

A significant advancement occurred in 1993 with the introduction of the intrapelvic approach by the Helsinki group. Hirvensalo et al. introduced a new anterior approach that required less dissection compared to the ilioinguinal approach while providing satisfactory long-term results [12].

A meta-analysis by Giannoudis et al. in 2005 reported that the majority of patients (up to 50%) were treated choosing posterior approaches, particularly the Kocher-Langenbeck approach. However, this analysis had limitations, notably the overrepresentation of certain fracture types, as many studies focused isolated posterior wall fractures and associated fracture types [9].

Regardless of these findings the Kocher-Langenbeck approach was the most frequently used approach throughout the 1990s and early 2000s, earning it the reputation as the ‘workhorse’ of acetabular fracture surgery [6, 47].

Recently, a fracture type–dependent, more individualized approach for the stabilization of acetabular fractures has been increasingly recommended [15, 43]. Various modifications of the Letournel approaches have been developed, along with several modifications of the intrapelvic approach.

The following modifications of the Kocher-Langenbeck approach were described:

- Surgical hip dislocation with trochanteric flip osteotomy [41].
- Gluteus maximus split approach [3].
- Modified Gibson approach [28, 29].
- Modified Kocher-Langenbeck approach [22, 23].
- Modified two-portal Kocher-Langenbeck approach [13].
- Modified Kocher-Langenbeck approach without detachment of the short external rotators [4, 38].

The modifications of the Kocher-Langenbeck approach according to Magu et al. and the two-portal variant according to Josten et al. avoided detachment of the short external rotators, thereby reducing muscular dissection [13, 22, 23]. However, despite these advantages, neither approach gained widespread adoption.

The modified Gibson approach, reintroduced by Moed et al., provides more extended access to the superior acetabular region [28, 29]. This extended access can also be achieved through surgical hip dislocation [7, 41, 42]. The latter technique allows additional access to the anterior acetabulum, making the variant of surgical hip dislocation with trochanteric flip osteotomy an evolving option as an extended Kocher-Langenbeck variant [41].

A recent literature review analyzed 8389 fractures from 8372 patients [16]. The Kocher-Langenbeck approach remains the ‘workhorse’ of acetabular fracture surgery, being performed in 48.7% of cases, with usage increasing to 54.8% during the last five years of the review period.

This overview will, therefore, analyze the value of the Kocher-Langenbeck approach, the Gibson variant, and the surgical hip dislocation approach according to Ganz (Ganz approach).

## Value of the Kocher-Langenbeck approach

The Kocher-Langenbeck approach involves a longitudinal Langenbeck incision, beginning above the greater sciatic notch and extending to the greater trochanter, with dissection of the gluteal muscles – a technique originally described for treating hip joint infections [49]. Theodor Kocher described a curved incision, starting at the posteroinferior corner of the greater trochanter, running across its posterosuperior tip, and passing obliquely in line with the fibers of the gluteus maximus muscle toward the posterior superior iliac spine [19]. These approaches were first combined by Osborne [32] and allowed reduction of fractures in the posterior region of the acetabulum under direct visualization.

Classical indications for the Kocher-Langenbeck approach primarily include fractures involving the posterior column area and many fractures with transverse fracture components [6].

The surgical steps of the Kocher-Langenbeck approach include:

- Slightly curved skin incision, starting just anterior to the posterior superior iliac spine, extending toward the tip of the greater trochanter, and following along the axis of the femur; the incision ends at the transition between the proximal and middle third of the thigh.
- Subcutaneous dissection in line with the skin incision.
- Sharp splitting of the iliotibial tract distally and blunt dissection of the fascia of the gluteus maximus muscle proximally in line with the skin incision.
- Identification and partial bursectomy of the trochanteric bursa and identification of the trochanteric branch of the medial circumflex femoral artery (MCFA).
- Identification of the quadratus femoris muscle (muscle fibers perpendicular to the axis of the femoral shaft).
- Palpating or visualization of the sciatic nerve posterior to the muscle belly usually without complete mobilization/dissection.
- Identification of the superior border of the quadratus femoris muscle.
- Identification of the interval between the distal triceps coxae and the quadratus femoris muscle containing the obturatorius internus tendon and the deep branch of the MCFA.
- Identification of the short external rotator muscles from distal to proximal (inferior gemellus, obturatorius, superior gemellus and piriformis).
- Sharp dissection of the triceps coxae muscle/tendons approx. 1 cm posterior from their insertions Respecting the course of the MCFA.
- Blunt mobilization of these muscles from the underlying capsule to the posterior border of the posterior column.
- Dissection of the piriformis muscle/tendon 1–2 cm posterior from its insertion.
- Capsulotomy as necessary under protection of the labrum.
- Extended dissection to the superior acetabular area by blunt mobilization of the gluteus medius and minimus muscles from the superior capsule and the supraacetabular periosteum.

Approach-related results are infrequently reported in the literature, often due to the challenges of inhomogeneous patient groups and varying acetabular fracture populations. Nonetheless, data from large patient collectives are available.

The Kocher-Langenbeck approach is not without risks. Letournel reported on Kocher-Langenbeck-related complications. Iatrogenic injuries of the superior gluteal artery and vein were observed in eight and six cases, respectively. 4.3% of patients developed postoperative hematomas. Iatrogenic sciatic nerve injuries were observed in 9.9% and the rate of deep infection after ORIF was reported to be 3.2% [20].

Rommens analyzed 60 consecutive patients with fractures of the posterior wall who were treated using the Kocher-Langenbeck approach [35]. The rate of postoperative sciatic nerve damage was 8.3%. A deep hematoma was observed in 3.3%, and an infection occurred in one patient (1.7%).

Tannast et al. reported on Matta’s data in 352 patients with acetabular fractures using the Kocher-Langenbeck approach [46]. The average blood loss was 800mL and the average operating time was 150 min.

In a recent analysis of 167 patients with different acetabular fractures treated with the Kocher-Langenbeck approach, the following complication rates were reported: infection rate 4,8%, nerve damage rate 15%, AVN rate 5,4%. Surgery time was 157 min and the median blood loss was 300mL [30].

Essence for clinical practice: The Kocher-Langenbeck approach remains the workhorse for posterior-based acetabular fracture patterns, demonstrating an overall low complication rate.

## Value of the (modified) Gibson approach

In 1950, Gibson described a modified Kocher-Langenbeck exposure to the hip joint, characterized by a more anterior orientation of the superior incision part [10].

Moed later introduced a further modification of this approach, avoiding the release of the gluteus medius and minimus tendons from the greater trochanter and limiting the extent of hip joint capsulotomy by performing a straight skin incision. Instead of splitting of the gluteus maximus muscle, as in the traditional Kocher-Langenbeck approach, Moed proposed dissection through the so-called Gibson interval between the gluteus maximus and tensor fasciae latae muscles, thereby reducing the risk of iatrogenic injury to the neurovascular supply to the anterior gluteus maximus muscle. The primary advantage is a more extended anterosuperior access to the hip joint [28, 29].

Moed described the modified Gibson approach with the patient in the prone position, detailing the following surgical steps:

- Straight skin incision from the mid-lateral thigh to the tip of the greater trochanter, extending proximally to the level of the iliac crest.
- Subcutaneous dissection in line with the skin incision.
- Identification of the anterior border of the gluteus maximus by visible branches from the inferior gluteal artery that perforate the fasciae latae.
- Fascial incision in line with the skin incision along the anterior border of the gluteus maximus muscle.
- Proximal separation of the gluteus medius muscle from the gluteal fascia.
- Postero-lateral retraction of the gluteus maximus muscle (if necessary distal release of its tendinous insertion) and anterior-medial retraction of the gluteal fascia/iliotibial tract.
- Identification of the sciatic nerve superficial to the quadratus femoris muscle.
- Release of the short external rotators and the piriformis muscle.
- If additional anterosuperior exposure is necessary, a bigastric trochanteric osteotomy has to be considered.

Moed reported approach-related outcomes in 16 patients with acetabular fractures [28, 29]. Heterotopic ossification occurred in 5 patients (all grade I or II), with no other approach-related complications reported.

While no other studies have focused on acetabular fracture outcomes using the isolated modified Gibson approach, several papers reported the combined use of the modified Gibson approach with the Ganz approach, which involves surgical dislocation of the hip with bigastric trochanteric osteotomy.

Essence for clinical practice: The modified Gibson approach is a modification of the Kocher-Langenbeck approach that potentially offers more extensive access to the superior acetabular region. However, there is a paucity of data on approach-related outcomes when using the isolated modified Gibson procedure.

## Value of the Ganz approach

Ganz and Siebenrock described the safe surgical hip dislocation technique based on the trochanteric flip osteotomy described by Mercati [26]. This technique extends the Kocher-Langenbeck approach to provide enhanced access to the superior, intraarticular, and anterior region of the acetabulum [7, 8, 41, 42]. This expanded approach can be performed following the Kocher-Langenbeck or, depending on the fracture type, the modified Gibson approach, while protecting the short external rotator muscles [24]. This technique facilitates complete visualization of the articular surface and improved exposure of the supraacetabular region. Furthermore, fractures with a transverse component can be more effectively reduced and fixed with an anterior column screw under direct visualization of both < the reduction and the implant position [50].

Results of the Bernese group involving 60 patients with various fracture types demonstrated an average surgical time of 204 min and an estimated blood loss of 1556 mL [45]. Four surgically-related complications were reported (6.7%): one case of iatrogenic superior gluteal nerve damage, one loss of reduction, one fracture nonunion, and one nonunion of the trochanteric osteotomy. Anatomical reduction was achieved in 93% of acetabular fractures.

Data focusing on specific fracture types (T-type fractures, pure transverse fractures, and associated transverse posterior wall fractures) showed an average operative time of 150 min and the mean blood loss of 1334 mL [24].

The Ganz procedure is associated with several advantages [18]:

- Low incidence of AVN: Compared to other approaches, it has a lower incidence of AVN, particularly when the short external rotators are preserved, though this is often not possible in acetabular fracture surgery [7, 17].
- Comprehensive visualization: Provides more or less 360° visualization of the acetabulum and femoral head.

However, potential complications include heterotopic ossifications in approximately one-third of cases [7, 14] and a non-union rate of 1–2% at the greater trochanter [7, 44].

A systematic review of Pipkin fractures, including type IV fractures (with acetabular fracture), reported an average blood loss of 491.89 mL with the Ganz approach. Complications occurred in 30–86% of cases, including 12% avascular necrosis, 25% heterotopic ossification, and 3,4% trochanteric osteotomy nonunion [17].

When compared to the Kocher-Langenbeck approach, the Ganz approach is associated with decreased abductor strength, lower rates of posttraumatic arthritis, and a slightly higher rate of heterotopic ossification in patients with posterior wall fractures [21]. The mean surgery time is slightly shorter with 134.7 min, with a modest increase in blood loss to 713.2 mL.

Essence for clinical practice: The Ganz surgical hip dislocation approach can be additionally performed after Kocher-Langenbeck or modified Gibson dissection and offers optimal 360° visualization of the acetabular surface and the femoral head with an overall low rate of significant complications.

## Patient positioning

Posterior approaches for acetabular fracture surgery can be performed with the patient in either the prone position, typically using a fracture table, or the standard lateral decubitus position on a radiolucent table. Both positions should allow for all necessary intraoperative views of the pelvis and acetabulum.

The prone position on a traction table offers several advantages. Gravity naturally reduces the femoral head, and 90° knee flexion releases tension to the sciatic nerve. Additionally, in fractures with a transverse component, digital access to the quadrilateral surface is more easily achieved compared to the lateral position, and interference from excessive abdominal pressure is minimized. However, a notable disadvantage is the requirement of an unscrubbed assistant to manipulate the table and adjust the hip position during surgery [5, 6].

The standard lateral decubitus position also has its benefits, particularly the ability to convert to a surgical hip dislocation with trochanteric flip osteotomy if necessary. This position also facilitates easier mobilization of the entire leg. However, there are potential disadvantages, such as the femoral head being displaced by gravity, which can push the fracture into a displaced position. This often necessitates lateral traction of the femoral head and neck using a Schanz screw. Additionally, there is a potential risk of sciatic nerve damage due to incomplete hip extension combined with only slight knee flexion [5, 6].

When comparing these different patient positions, no advantages or disadvantages have been identified regarding quality of reduction [5, 31]. However, the prone position has been associated with a higher rate of postoperative infections and revision surgeries and, possibly due to longer positioning times, a potential risk of nosocomial infection was proposed [31].

In our experience, the standard lateral decubitus position is preferred, primarily because it allows for the option of extending the procedure to include a surgical hip dislocation in selected fractures, which is not feasible in the prone position.

Recent analysis has shown that lateral positioning is associated with significantly shorter surgical times and a lower incidence of iatrogenic sciatic nerve injuries, without significant differences in blood loss, heterotopic ossification, or infection rates [37].

In a study analyzing the rate of sciatic nerve injuries in 273 patients, the prone position was significantly associated with a higher risk of sciatic nerve injury during surgery [2]. In contrast, a larger study involving 644 patients with acetabular fractures found no significant difference in sciatic nerve injury rates between prone (3.1%) and lateral (3.3%) positions. Identified risk factors for sciatic nerve injury included the transverse fracture pattern (odds ratio 3.0) and the individual surgeon [39].

Essence for clinical practice: Posterior approaches can be successfully performed in either the prone or lateral decubitus position, both leading to satisfactory outcomes. The primary advantage of the lateral decubitus position is the flexibility to convert to a surgical hip dislocation with trochanteric flip osteotomy (Ganz approach) if necessary.

## Exposure

The classical Kocher-Langenbeck approach provides direct visualization of the entire posterior column, posterior wall and part of the supraacetabular region. It also allows palpation of parts of the inner surface of the true pelvis through the suprapiriform foramen. The modified Gibson variant offers similar access, with potentially improved anterior-superior exposure [6, 29, 47]. The bigastric trochanteric osteotomy with surgical hip dislocation enables near-total exposure of the acetabular roof and nearly complete direct visualization of the articular surface [7, 41, 42].

However, the Kocher-Langenbeck approach may provide limited exposure in certain posterior fracture types, especially high posterior wall fractures with anterior-superior extension [1, 20, 41]. The modified Gibson and Ganz approaches formally allow for greater anterior and cranial dissection.

Mitchell et al. conducted a cadaver study to compare the anterior and cranial exposure offered by the Kocher-Langenbeck and the Gibson approaches, with and without additional trochanter flip osteotomy (Ganz modification) [27]. The study found an overall comparable access between the two approaches. The modified Gibson approach achieved a mean surface area was 9.2 cm^2^ without and 13.3 cm^2^ with trochanteric osteotomy, while the Kocher-Langenbeck approach yielded 8.9 cm^2^ without and 12.7 cm^2^ with trochanteric osteotomy. The difference between the two approaches was minimal (0.3 cm^2^ surface area without and 0.6 cm^2^ with trochanteric osteotomy). In only 2 of eight cadavers, the modified Gibson approach allowed for a significant increase in anterior exposure compared to the Kocher-Langenbeck approach. However, performing a trochanteric osteotomy consistently increased the anterior exposure of both approaches, yielding an average increase of 1.5 cm with the modified Gibson approach and 1.6 cm with the Kocher-Langenbeck approach.

**Fig. 1:**
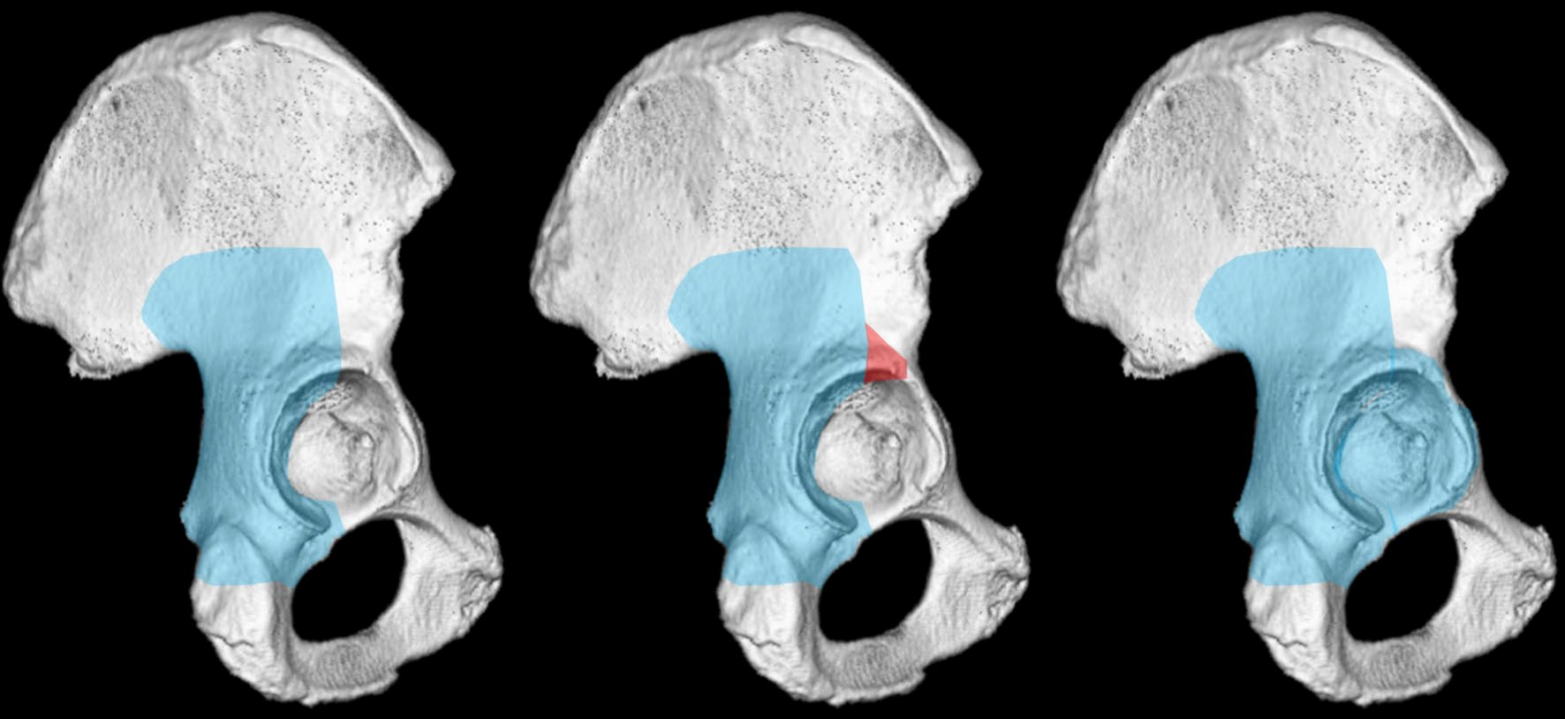
Comparison of surgical exposures provided by various posterior approaches: Kocher-Langenbeck approach (left), modified Gibson approach (as detailed in references [28, 29]) (middle), and Ganz approach (right)

A subsequent cadaver analysis by Vemulapalli et al. corroborated these findings [48]. The study compared the surface access area of the classical Kocher–Langenbeck approach, the Kocher-Langenbeck approach after gluteus minimus debridement, and the modified Gibson approach. The modified Gibson approach provided an average surface area of 15.4 cm^2^, while the Kocher-Langenbeck approach yielded 22.5 cm^2^, which increased to 35.6 cm^2^ after gluteus minimus debridement. These results align with Moed’s report, which graphically illustrates a marginal improvement in visual access with the modified Gibson approach (Fig. 1) [28, 29].

A recent cadaveric study assessed the extent to which a trochanteric slide osteotomy enhances the exposure provided by the Kocher-Langenbeck approach. The study found that performing a trochanteric slide osteotomy increased the mean acetabular surface area exposed by the Kocher-Langenbeck approach from 27.66 cm^2^ to 41.82 cm^2^, representing a 51.2% increase in exposure [33]. The study findings suggest that while both exposures allow adequate palpation and visualization of the superior acetabulum, the addition of a trochanteric osteotomy significantly enhances visual access to the anterior-superior portions of the acetabulum.

**Fig. 2:**
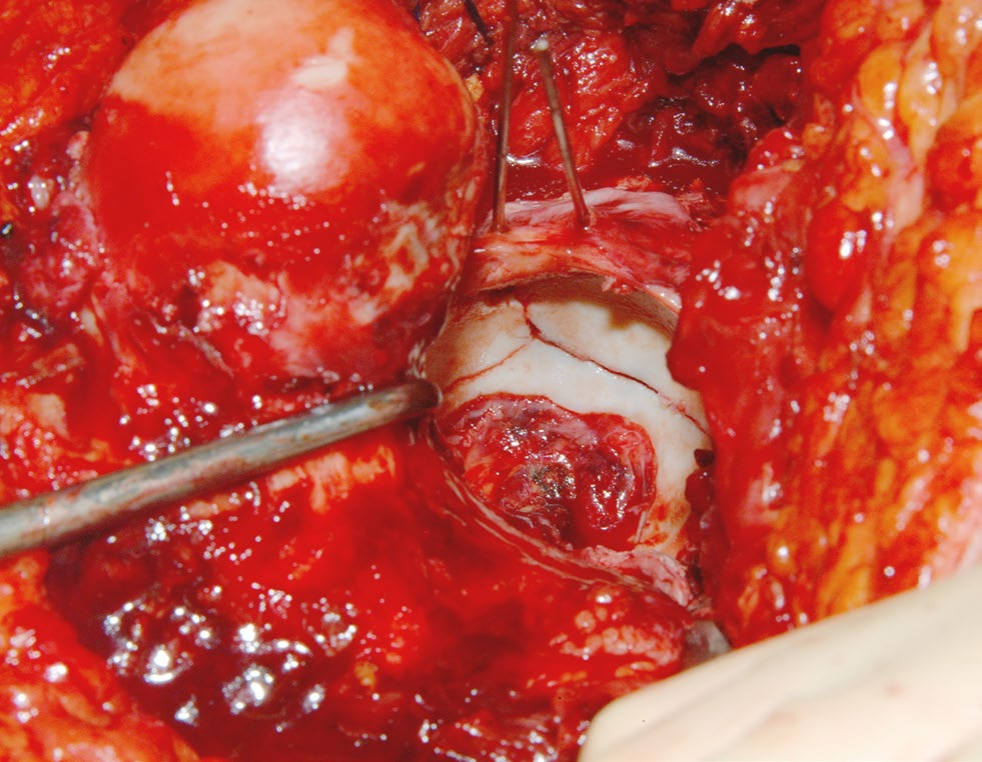
Intraoperative exposure achieved using the Ganz approach with surgical hip dislocation. This technique provides comprehensive visualization of the acetabulum, particularly enhancing access to the anterior and superior walls, as well as the entire intraarticular surface

Essence for clinical practice: Dissection analyses confirmed that both the Kocher-Langenbeck and Gibson approach provide sufficient visualization of the posterior and superior acetabulum, with the Kocher-Langenbeck approach potentially offering a slight advantage. However, the Ganz approach remains superior for achieving comprehensive acetabular exposure, particularly in cases requiring full access to the anterior-superior acetabulum or to the articular surface (Fig. 2).

## Conclusion

The classical posterior approaches for acetabular fracture surgery, such as the Kocher-Langenbeck and modified Gibson approaches, provide adequate visualization of the posterior and superior acetabulum. In cases of complex and comminuted posterior acetabular fractures, the extended posterior approach, specifically the Ganz approach with bigastric trochanteric osteotomy and safe surgical hip dislocation, offers optimal exposure by enabling a comprehensive 360° view of both the acetabulum and femoral head. While approach-related outcomes are not extensively reported, the available data suggest a low incidence of approach-related complications, with heterotopic ossification being the most common. No relevant difference has been observed between lateral decubitus and prone positioning in terms of surgical outcomes. Thus, the choice of posterior surgical approach and patient positioning should be guided by the surgeon’s expertise and preference, tailored to the specific fracture pattern and patient needs.

## Data Availability

A data availability statement is not applicable to this article because this paper is no original research generating original data but rather a clinical review article. Thus, there cannot be any publicly archived datasets analysed or generated during the study.
